# Learning to Work With Chronic Health Conditions: How Time Since Diagnosis Affects Older Workers' Vitality and Worries

**DOI:** 10.1093/geroni/igab046.2129

**Published:** 2021-12-17

**Authors:** Anushiya Vanajan, Ute Bültmann, Kène Henkens

**Affiliations:** 1 Netherlands Interdisciplinary Demographic Institute (NIDI), The Hague, Netherlands, Den Haag, Zuid-Holland, Netherlands; 2 University Medical Center Groningen (UMCG), Groningen, Groningen, Netherlands; 3 Netherlands Interdisciplinary Demographic Institute (NIDI), The Hague, Zuid-Holland, Netherlands

## Abstract

Background. Chronic health conditions (CHCs) pose stark detrimental effects on the health and abilities of older workers. The extent of these effects depend on the CHC, the time since its diagnosis and the type of health measure: a rarely explored combination of heterogeneities. Objective. This study examined how four existing and newly diagnosed CHCs influences older workers’ vitality and worries about enduring physically and mentally until retirement age. Method. Data from two waves of the NIDI Pension Panel survey conducted in the Netherlands in 2015 and 2018 were used. We analyzed a sample of 1,894 older workers between the ages of 60-62 years at wave 1 using conditional change ordinal least square regression models. Results. Having a CHC at wave 1 was associated with lower levels of vitality and higher levels of worries at wave 2. These effects of CHCs on vitality and worries were much larger for older workers who were newly diagnosed with CHCs compared to those who experienced CHCs for longer. Intriguingly, the new diagnosis of physically disabling conditions increased worries about physical endurance at wave 2, while the new diagnosis of mentally disabling conditions increased worries about mental endurance at wave 2. Conclusion. By distinguishing the effects of four existing and newly diagnosed CHCs on vitality and worries, this study allows the identification of vulnerable groups of older workers. The findings may inform work accommodations and interventions which could improve both the quality and sustainability of work lives, while promoting healthy ageing of older workers.

